# Synthetic ACTH for Treatment of Glomerular Diseases: A Case Series

**DOI:** 10.1177/20543581211066979

**Published:** 2022-01-05

**Authors:** Arenn Jauhal, Bhanu Prasad, Mathieu Rousseau-Gagnon, Gabriel Ouellet, Michelle A Hladunewich

**Affiliations:** 1 Division of Nephrology, Department of Medicine, University of Toronto, Toronto, ON, Canada; 2 Section of Nephrology, Department of Medicine, Regina General Hospital, Regina, SK, Canada; 3 Division of Nephrology, CHU de Québec-Université Laval, Québec City, QC, Canada

**Keywords:** synthetic adrenocorticotropic hormone, ACTH, Tetracosactide, Synacthen Depot, glomerulonephritis, remission, adverse events

## Abstract

**Rationale:**

Synthetic adrenocorticotropic hormone (Tetracosactide) has been used in the treatment of refractory glomerular diseases. Literature surrounding the use of this medication is limited to small case series and there is conflicting data on the rate of adverse events associated with this medication.

**Presenting concerns of the patient:**

Glomerulonephritis not in remission after at least 6 months of treatment with conservative care. Stable doses of concurrent immunosuppression were permitted.

**Diagnoses:**

Membranous nephropathy, IgA nephropathy, minimal change disease, and focal and segmental glomerulosclerosis.

**Intervention:**

Intramuscular synthetic adrenocorticotropic hormone (Tetracosactide, Synacthen Depot) with doses of either 1 mg weekly or 1 mg twice weekly.

**Outcomes:**

Five of 12 patients had at least a partial remission with Tetracosactide. Median time to response was 6 months for responders. Five of the 12 patients had adverse events documented, 2 of which led to treatment discontinuation. No patients with focal and segmental glomerulosclerosis responded to treatment.

**Lessons Learned:**

Higher rate of adverse events than previously reported with synthetic adrenocorticotropic hormone and uncertain treatment efficacy.

## Abrégé

**Justification:**

L’hormone adrénocorticotrope synthétique (tétracosactide) a été utilisée pour le traitement des maladies glomérulaires réfractaires. La littérature portant sur l’utilisation de ce médicament est limitée à de petites séries de cas et les données sur le taux d’événements indésirables associés à ce médicament sont contradictoires.

**Présentation des cas:**

Glomérulonéphrites qui ne sont pas en rémission après un minimum de six mois de traitement conservateur. Des doses stables de traitement immunosuppresseur concomitant étaient autorisées.

**Diagnostics:**

Néphropathie membraneuse, néphropathie à IgA, néphropathie à lésion glomérulaire minime, hyalinose segmentaire et focale.

**Interventions:**

Des doses soit de 1 mg par semaine soit de 1 mg deux fois par semaine d’hormone adrénocorticotrope synthétique (Tetracosactide, Synacthen Depot) administrées par voie intramusculaire.

**Résultats:**

Cinq patients sur douze ont connu au moins une rémission partielle avec le tétracosactide. Le délai de réponse médian était de six mois pour les patients qui répondaient au traitement. Cinq des douze patients ont eu des réactions indésirables documentées, dont deux ont entraîné l’arrêt du traitement. Aucun des patients présentant une hyalinose segmentaire et focale n’a répondu au traitement.

**Enseignements tirés:**

Un taux de réactions indésirables plus élevé que celui rapporté précédemment avec l’hormone adrénocorticotrope synthétique et une efficacité incertaine du traitement.

## What was known before

Natural and synthetic adrenocorticotropic hormone (ACTH) formulations have been used in the treatment of glomerulonephritis, with the majority of literature surrounding the natural form. Case series of synthetic ACTH use in glomerulonephritis have included small populations, ranging from 2 to 14 patients. The reporting of adverse events has been inconsistent.

## What this adds

Our case series found a higher rate of adverse events than previously reported with synthetic ACTH. Appropriate assessment of comorbidities and immunosuppression history should be made when considering this treatment.

## Introduction

Managing glomerulonephritis (GN) remains challenging, often requiring medications with a high risk of adverse events and unclear efficacy. One such treatment is adrenocorticotropic hormone (ACTH) therapy. Adrenocorticotropic hormone acts as an agonist of the melanocortin system by binding to all 5 melanocortin receptors.^1^ These melanocortin receptors have been found in many cells including podocytes, glomerular cells, and immune cells.^1^ Possible effects on podocytes include stabilization, apoptosis reduction, downregulating inflammation, and decreased binding of complement proteins.^1^

There are 2 forms of ACTH, natural and synthetic. The natural form (H.P. Acthar gel) is the pharmaceutical derivative available for treatment in the United States. It was one of the first therapies approved by the Food and Drug Administration (FDA) for the treatment of nephrotic syndrome in 1952.^2^ The natural ACTH was canceled post market in 1997 in Canada.^3^ The synthetic ACTH, also known as Tetracosactide (Synacthen Depot), is the only one currently available in Canada.^3^ The majority of the literature for GN involves natural ACTH.^4-8^ The cases series for use of synthetic ACTH in GN are small, ranging from 2 to 14 patients.^9,10^

A recent systematic review suggested that ACTH may be used in a variety of glomerular diseases; however, that review consisted of cases primarily treated with the natural form.^11^ There seems to be a large variation in the adverse event reporting between studies. Some case series reported infrequent and mild side effects, whereas others found very high rates of adverse events (including serious adverse events) associated with ACTH.^12^ Concern about greater rates of adverse events and only modest efficacy suggests further evidence is needed for synthetic ACTH in use in glomerular diseases. To assess the experience with synthetic ACTH for use in GN in our Canadian centers, we conducted a multicenter case series of patients with IgA nephropathy, membranous nephropathy, focal and segmental glomerulosclerosis, and minimal change disease that were treated with synthetic ACTH.

## Methods

We included all patients treated with intramuscular Tetracosactide (Synacthen Depot) at 3 tertiary care hospitals in Saskatchewan, Ontario, and Quebec from 2013 to 2019. Research ethics approval and data sharing agreements were obtained from each institution.

We collected data by manual chart review. Stable doses of concurrent immunosuppression for at least 6 months were required before Tetracosactide initiation. The main outcome was at least a partial remission of their GN according to KDIGO guidelines.^13^ Remission for IgA nephropathy was classified as proteinuria less than 1 g/d. For membranous nephropathy, focal and segmental glomerulosclerosis, and minimal change disease, partial and complete remission were classified as proteinuria less than 3.5 g/d and less than 0.3 g/d, respectively. Laboratory measurements were monitored during ACTH use and for at least 6 months after cessation of therapy to determine whether a remission occured.

## Results

We included 12 patients across the 3 participating sites (Table 1). The median age was 43 years (range, 24-68 years); there were 7 males and 5 females. Five of the patients had IgA nephropathy, 3 patients had membranous nephropathy, 3 patients had FSGS disease, and 1 patient had minimal change disease. The median serum creatinine at the time of ACTH initiation was 124 µmol/L (range, 60-229 µmol/L), and median proteinuria was 6.2 g/d (range, 1.05-15.7 g/d).

**Table 1.** Summary of Patients Who Received Tetracosactide (Synacthen Depot) for Treatment of Their Glomerular Disease.

	Dx	Age	Sex	Comorbidities	Baseline Cr (µmol/L)	Baseline proteinuria (g/d)	Prior therapy	ACTH Dose (duration in months)	Remission	Finał proteinuria g/d (if remit)	Time to remission (months)
1	MCD/DM	53	M	DM, HTN, obesity, smoking	89	7.75	CNI, Cyclo, Ritux	1 mg weekly (18)	**Y**	1.37	6
2	MN	48	M	HTN, obesity	95	15.0	S, Cyclo, Ritux	1 mg weekly (12)	**Y**	0.16	18
3	IgA	41	F	—	108	9.58	S, Cyclo (concurrent)	1 mg weekly (10)	N	—	—
4	IgA	44	F	HTN	138	4.10	S, cyclophos	1 mg weekly (26)	**Y** ^a^	0.88	10
5	IgA	24	F	—	60	2.57	MMF, S	1 mg weekly (11)	**Y**	0.78	2
6	IgA	33	F	—	60	1.05	None	1 mg twice weekly	N	—	—
7	FSGS	28	M	HTN	229	2.91	S, CNI, Ritux	1 mg weekly	N^b^	—	—
8	IgA	31	M	HTN	80	14.38	S, Cyclo (concurrent)	1 mg twice weekly	N	—	—
9	FSGS	58	M	DM, HTN, smoking	228	4.9	CNI, Ritux	1 mg weekly	N	—	—
10	FSGS	41	F	—	194	15.7	S, CNI, MMF, PLEX	1 mg twice weekly	N	—	—
11	MN	68	M	—	154	5.2	CNI (concurrent)	1 mg twice weekly	N	—	—
12	MN	66	M	—	163	7.16	S, MMF, CNI (concurrent)	1 mg twice weekly	**Y** ^c^	3.0	6

*Note.* Final proteinuria only displayed for those who had at least a partial remission. Dose displayed in table is the maximum dose, and not necessarily the exact dose during the time duration indicated. Patients may have started weaning during the duration (months) indicated. Definitions of CR/PR are based on the KDIGO 2012 guidelines. IgA CR/PR defined as less than 1 g/d proteinuria. MN/FSGS/MCD PR less than 3.5 g/d and CR less than 0.3 g/d. ACTH = adrenocorticotropic hormone; DM = diabetes; HTN = hypertension; MCD = minimal change disease; MN = membranous nephropathy; S = steroids; CNI = calcineurin inhibitor; Ritux = Rituximab; MMF = mycophenolic acid; Cyclo = cyclophosphamide; CR = complete remission; FSGS = focal and segmental glomerulosclerosis; KDIGO = Kidney Disease Improving Global Outcomes; PLEX = plasmapheresis; PR = partial remission.

a Patient relapsed on discontinuation.

b Medication discontinued due to side effects and cost.

c Medication discontinued due to side effects.

Each patient had received a variety of immunosuppressive regiments before being placed on Tetracosactide. The most common previous and concurrent therapies were cyclophosphamide (n = 5), corticosteroids (n = 5), calcineurin inhibitors (CNIs; n = 3), and rituximab (n = 3). Four patients were on concurrent immunosuppression at the time of ACTH treatment.

Five of the patients had at least a partial response to Tetracosactide, and 7 patients did not. Responders had a median time to response of 6 months (range, 2-18 months). One patient had an initial response, but discontinued Tetracosactide due to significant adverse events, and therefore, their response was not sustained (patient 12). Patient 3 did not have a response to 4 months of Tetracosactide and then cyclophosphamide was added; proteinuria then improved to 0.6 g/d by 6 months, but this response was attributed to cyclophosphamide rather than ACTH.

Only patient 7 had a history of steroid responsiveness. Patients 3-6, 8, and 12 did not have a significant response to prednisone therapy. Patients 1-2 and 9-11 did not have documentation of response to steroids.

Another patient had to be discontinued from Tetracosactide due to side effects, and although their proteinuria may have decreased, they did not meet the requirements for complete or partial remission (patient 7).

Five of the 12 patients had adverse events documented that were felt to be related to Tetracosactide (Table 2); 2 of these patients were on concurrent immunosuppression treatment. Edema, hypertension, hypokalemia, cushingoid features, and mood issues were experienced by more than 1 patient. Infections were also reported, including *Clostridium difficile*, community-acquired pneumonia, and *Mycobacterium avium* complex; the latter 2 were deemed serious adverse events as they required hospitalization. Patient 7 discontinued treatment due to both adverse events and medication cost. Patient 12 discontinued treatment due to adverse events.

**Table 2.** Adverse Events Associated With Tetracosactide (Synacthen Depot).

Case	Adverse events
1	Not documented
2	Not documented
3	Not documented
4	Not documented
5	Not documented
6	Feeling generally unwell, Cushingoid features (only on 1 mg twice weekly dose)
7	Nausea, hiccups, abnormal bowel movements, rashes
8	Not documented
9	Not documented
10	Cushing syndrome, DM on insulin, HTN, hypokalemia, severe insomnia, anxiety, difficulty concentrating, CAP, C. diff
11	Edema, hypertension, hypokalemia, dyslipidemia, MAC infection
12	Edema, SOB, hypokalemia, fatigue, low mood

*Note.* DM = diabetes mellitus; HTN = hypertension; CAP = community-acquired pneumonia; C. diff = *Clostridium difficile* infection; MAC = *Mycobacterium avium* complex; SOB = shortness of breath.

## Discussion

In our case series, 5 of 12 patients with glomerular disease had a complete or partial remission after treatment with Tetracosactide. Of the responders, 2 had membranous nephropathy, 2 had IgA nephropathy, and 1 had minimal change disease overlapping with diabetic nephropathy. No patients with FSGS disease responded to treatment. The median time to response was 6 months (range, 2-18 months). Five patients had adverse events associated with Tetracosactide, 2 of which led to treatment discontinuation.

The types of GN that responded to Tetracosactide appear to be concordant with previous literature.^3^ A recent systematic review by Chakraborty et al found that remission rates for membranous nephropathy, IgA nephropathy, and minimal change disease were 70%, 46%, and 79%, respectively.^11^ Suggestion that IgA nephropathy is less responsive to ACTH therapy may be due to a paucity of evidence.^14^ The overall lower response rate in our study may be due to the different formulations of ACTH used in the literature and our small sample size. One of the 5 patients (patient 12) with remission on Tetracosactide was on concurrent immunosuppression. Although multitargeted therapy and not ACTH alone may have led to a remission, given this patient had been on stable doses of CNI without prior remission, it was felt that ACTH did contribute to the remission.

We encountered a higher rate of adverse events associated with Tetracosactide than other studies.^11^ Although the majority of adverse event rate reporting for both synthetic and natural ACTH reported in the literature is low, there have been studies reporting an adverse event rate of 95% and serious adverse events ranges as high as 25%.^11,12^ The majority of the adverse events in our study included Cushing syndrome, hypertension, edema, dyslipidemia, and mood disturbances. Serious adverse events included major infections requiring hospital admission. There were also side effects unique to ACTH reported, such as hypokalemia. Although age, comorbidities, and immunosuppression history play an important factor in the development of adverse events with any treatment for glomerular disease, the rate and type of side effects noted in our case series is difficult to ignore. Two of the 5 patients with adverse events were on concurrent immunosuppression but were tolerating their treatment with stable doses prior to initiation of ACTH. Therefore, the side effects were felt to be due to synthetic ACTH therapy, but confounding is possible.

Synthetic ACTH was used for all 12 of the patients (Atnahs Pharma UK Limited), which is the only formulation available in Canada. Although there are no studies suggesting that natural ACTH is superior to synthetic ACTH, or has a better adverse event profile, there are pharmacological studies that suggest different properties between the 2 forms.^15^ The C-terminus that is missing in the synthetic ACTH originally had uncertain biological function, but evidence shows it confers stability, enhances activity, and contributes to lipostasis.^15-18^ These may account for different results when using ACTH treatment for GN. Prospective clinical trials are needed to determine the differences between the natural and synthetic ACTH, however, approval for natural ACTH has not been sought by the pharmaceutical company in Canada. The systemic review by Chakraborty et al reflects an emergence of natural ACTH use in the United States in recent years.^11^ In Ontario, the cost of Tetracosactide (Synacthen Depot) is $680 per 1 mg, as per the Ontario Drug Benefit (ODB) formulary.^19^ This reflects the cost burden for patients having to try alternative therapies for their refractory GN.

Our study is limited by the small number of patients and lack of comparator group. Many of the patients had received and/or were on multiple immunosuppressive medications. In addition, it is difficult to know how the specific timing of these other therapies impacts the effects of ACTH. Therefore, any treatment effect was difficult to attribute to Tetracosactide alone. However, our study is among the largest case series of GN patients treated with Tetracosactide (Synacthen depot) and adds important information for patients with glomerular disease considering treatment with synthetic ACTH. Larger cohort studies with a comparator group are needed to appropriately assess the efficacy and adverse events for the use of synthetic ACTH in glomerular disease.

In summary, treatment decisions surrounding the use of synthetic ACTH for glomerular diseases are based on small case series. Natural ACTH is not available in Canada; therefore, synthetic ACTH is the only option. Although synthetic ACTH was effective in inducing complete and partial remissions in some of the cases, our series supports the conclusion that adverse events may be underreported with this medication, including serious infections. Synthetic ACTH use may be considered in patients with membranous nephropathy, IgA nephropathy, or minimal change disease particularly in patients with contraindications to other therapies and/or evidence of refractory disease. Appropriate assessment of comorbidities and immunosuppression history should be made prior to initiation of treatment to accurately assess risk of adverse events and implement any necessary mitigation measures. We do not recommend using synthetic ACTH for FSGS disease. Natural ACTH may have a better side effect profile for patients with GN, but further studies are needed to confirm this difference between these two formulations.
